# *Pseudomonas aeruginosa* Isolates from Spanish Children: Occurrence in Faecal Samples, Antimicrobial Resistance, Virulence, and Molecular Typing

**DOI:** 10.1155/2018/8060178

**Published:** 2018-06-11

**Authors:** Lidia Ruiz-Roldán, Alba Bellés, Jessica Bueno, José Manuel Azcona-Gutiérrez, Beatriz Rojo-Bezares, Carmen Torres, F. Javier Castillo, Yolanda Sáenz, Cristina Seral

**Affiliations:** ^1^Área de Microbiología Molecular, Centro de Investigación Biomédica de La Rioja (CIBIR), Logroño, Spain; ^2^Servicio de Microbiología, IIS Aragón, Hospital Clínico Universitario Lozano Blesa, Zaragoza, Spain; ^3^Departamento de Diagnóstico Biomédico, Laboratorio de Microbiología, Hospital San Pedro, Logroño, Spain; ^4^Área de Bioquímica y Biología Molecular, Universidad de La Rioja, Logroño, Spain; ^5^Departamento de Microbiología, Facultad de Medicina, Universidad de Zaragoza, Zaragoza, Spain

## Abstract

*Pseudomonas aeruginosa* is a major opportunistic human pathogen, responsible for nosocomial infections and infections in patients with impaired immune systems. Little data exist about the faecal colonisation by* P. aeruginosa* isolates in healthy humans. The occurrence, antimicrobial resistance phenotype, virulence genotype, and genetic lineages of* P. aeruginosa* from faecal samples of children from two different Spanish regions were characterised. Seventy-two* P. aeruginosa* were isolated from 1,443 faecal samples. Low antimicrobial resistance levels were detected: ceftazidime (8%), cefepime (7%), aztreonam (7%), gentamicin (3%), ciprofloxacin (1%), and imipenem (1%); susceptibility to meropenem, amikacin, tobramycin, levofloxacin, and colistin. Four multidrug-resistant strains were found. Important differences were detected between both geographical regions. Forty-one sequence types were detected among the 48 tested strains. Virulence and quorum sensing genes were analysed and 13 virulotypes were detected, being 26* exoU*-positive strains. Alteration in protein OprD showed eight different patterns. The unique imipenem-resistant strain showed a premature stop codon in OprD. Intestinal colonisation by* P. aeruginosa*, mainly by international clones (as ST244, ST253, and ST274), is an important factor for the systemic infections development and the environmental dissemination. Periodic active surveillance is useful to identify these community human reservoirs and to control the evolution of antibiotic resistance and virulence activity.

## 1. Introduction

*Pseudomonas aeruginosa* is a ubiquitous Gram-negative bacteria present in many diverse environmental settings, including different living sources, such as animals and humans. The ability of* P. aeruginosa* to survive on minimal nutritional requirements and to tolerate a variety of physical conditions has allowed this organism to persist in both community and hospital settings [1]. This species is a major opportunistic human pathogen, responsible for worldwide nosocomial infections with increasing medical and veterinary importance [1] and causing infections mostly in patients with impaired immune systems [2].* P. aeruginosa* shows high intrinsic resistance to antibiotics and has an extraordinary capacity to acquire new resistance mechanisms [3–5]. The increasing prevalence of multidrug-resistant (MDR)* P. aeruginosa* isolates is a global health problem, because of the limitation in clinical treatment options [5, 6].

This microorganism is seldom a member of the normal microbiota in humans. Colonisation rates in the range of 2.6 to 24% have been reported in human faecal samples [1, 3, 7]. Nevertheless, other studies show a high prevalence of* P. aeruginosa* in small intestinal and faecal samples from irritable bowel syndrome patients [7]. Relatively little data exist so far about the faecal colonisation by* P. aeruginosa* isolates in healthy humans, as well as about their antimicrobial resistance and virulence characteristics [1, 3, 7, 8]. Children tend to be exposed to more disease-causing bacteria through diary activities such as childcare and mouthing behaviours; they are more vulnerable to bacterial illness than adults, and they develop many nonfatal bacterial infections that require antimicrobial treatments, whereas no descriptions exist about the faecal carriage and characterisation of* P. aeruginosa* isolates from children. For that reason, the aim of this study was to determine the occurrence, the antimicrobial resistance phenotypes, the virulence genotypes, and the genetic lineages of* P. aeruginosa* in faecal samples of children from two Spanish regions (La Rioja and Aragón).

## 2. Material and Methods

### 2.1. Bacterial Strains

A total of 966 faecal samples of children were recovered in the Hospital Clínico Universitario Lozano Blesa (HLB) (Zaragoza, Aragón, Spain) from June to October 2013 and 477 samples in the Hospital San Pedro (HSP) (Logroño, La Rioja, Spain) from June to August 2014. La Rioja and Aragón are close regions but have different epidemiology settings. All children under 15 years old, who attended the hospital primary cares with gastroenteritis, food allergies, or disorders and with a microbiological study of their stools, were included in the study. The recruitment was the same in both hospitals. Samples were recovered from unique patients without repeated sampling from same patients. Samples were streaked onto cetrimide agar plates. After incubation at 37°C during 24h, one colony per sample with* P. aeruginosa* morphology was selected. Identification of the isolates was performed by classical biochemical methods and MicroScan® WalkAway (Siemens) microdilution system.

### 2.2. Antimicrobial Susceptibility Testing

*In vitro* antimicrobial susceptibility testing was performed by MicroScan WalkAway (Siemens) microdilution system following the Clinical and Laboratory Standards Institute (CLSI) guidelines [9]. Thirteen antimicrobial agents were tested, including (range of tested Minimal Inhibitory Concentration in *μ*g/mL, MIC): ticarcillin (8-32), piperacillin-tazobactam (8/4-64/4), aztreonam (1-16), cefepime (1-16), ceftazidime (1-32), meropenem (1-8), imipenem (1-8), tobramycin (2-8), gentamicin (1-8), amikacin (8-32), levofloxacin (1-4), ciprofloxacin (0.5-2), and colistin (2-4). Extended-spectrum-beta-lactamase (ESBL), metallo-beta-lactamase (MBL), and class A carbapenemase phenotypes were determined by double-disc synergy tests [10–12].

### 2.3. Molecular Typing

The clonal relationship among the recovered isolates was determined by pulsed-field gel electrophoresis (PFGE) with* SpeI* restriction enzyme to digest genomic DNA [3].

Multilocus sequence typing (MLST) for* P. aeruginosa* was performed by PCR and sequencing of seven housekeeping genes (*acsA, aroE, guaA, mutL, nuoD, ppsA, *and* trpE*) [13] (see Table S1 Supplementary Material). Allelic profiles and sequence types (STs) were compared with the PubMLST database (http://pubmlst.org/paeruginosa/). New STs were submitted to the PubMLST website.

### 2.4. Virulence Marker Genes

The following virulence and quorum sensing genes were analysed by PCR [14]:* exoU, exoS, exoY, exoT, lasA, lasB, exoA, aprA, rhlAB, rhlI, rhlR, lasI, *and* lasR *(see Table S1 Supplementary Material).

### 2.5. Detection and Characterisation of Integrons

The presence of genes encoding type 1, 2, and 3 integrases, and 3′-conserved segment of class 1 integrons (*qacE*Δ1*+sul1*) was studied by PCR. The characterisation of class 1 integron variable regions was carried out by PCR mapping and sequencing [15] (see Table S1 Supplementary Material).

### 2.6. Characterisation of OprD Porin

The amino acid changes of the porin protein OprD were analysed by PCR and sequencing in all* P. aeruginosa* strains [16, 17] (see Table S1 Supplementary Material). The mutations were determined by comparison with the sequence of the control strain* P. aeruginosa* PAO1 (GenBank accession number AE004091).

The outer membrane proteins of selected strains were obtained and visualized by SDS-PAGE (Sodium Dodecyl Sulphate-PolyAcrylamide Gel Electrophoresis) in a Bio-Rad apparatus (Mini-Protean II) and were stained with Coomassie Brilliant Blue as previously described [18]. The* P. aeruginosa* PAO1 and PAO1 lacking-OprD were included as control strains.

## 3. Results

### 3.1. *P. aeruginosa* Isolates and Antimicrobial Susceptibility

A total of 72* P. aeruginosa* were isolated from 1,443 faecal samples of children (5%): 42 isolates were obtained in HLB (4.3%) and the 30 remaining ones in HSP (6.3%).* Campylobacter, Salmonella, Giardia*, adenovirus, or rotavirus were the microorganisms involved in the diagnosis of 12 out of the 72* P. aeruginosa*-positive children. Only 10 of the 72 children showed any underlying disease (number of children): atopic dermatitis (2), weight delay (2), lactose intolerance (2), Williams syndrome (1), anorexia (1), cystic fibrosis (1), or psychomotor disturbance/delay (1). No relation was detected between the clinical background of the children and the* P. aeruginosa* isolates (see Table S2 Supplementary Material).

All isolates from HSP were susceptible to all antimicrobial agents tested, with the exception of one isolate that was ceftazidime resistant. On the other hand, the 42* P. aeruginosa* isolates from HLB showed the following resistance percentages to cefepime, 11.9%; piperacillin-tazobactam, 11.9%; ticarcillin, 11.9%; aztreonam, 11.9%; ceftazidime, 11.9%; gentamicin, 4.7%; imipenem, 2.4%; ciprofloxacin, 2.4% and were susceptible to meropenem, amikacin, tobramycin, levofloxacin, and colistin. Only 4 multidrug-resistant isolates were found (Pc31, Pc33, Pc40, and Pc43). None of the 72 isolates showed class A carbapenemase, MBL, or ESBL phenotypes.

Only two out of the 72* P. aeruginosa*-harbouring children had received antibiotic treatment in the three months previous to sampling: i.e., one cystic fibrosis child (individual 44) who took amoxicillin-clavulanic acid due to methicillin-susceptible* S. aureus* presence and another child (individual 60) who suffered a urine infection and took meropenem, whereas the* P. aeruginosa* isolated from both patients were piperacillin-tazobactam-resistant and susceptible, respectively.

### 3.2. Clonal Relationship and Molecular Typing

Sixty-eight different PFGE patterns were detected among the 72* P. aeruginosa:* 39 different PFGE patterns among the 42 isolates from HLB, and 29 among the 30* P. aeruginosa* isolates from HSP. No relationship was found among strains from HLB and those from HSP.

The sequence types (ST) were determined among 48* P. aeruginosa* strains using MLST method. Forty-one different sequence types were observed, five of them (ST2221, ST2222, ST2223, ST2241, and ST2242) being firstly described in this study and were annotated in the MLST database. Six ST were repeated more than once: ST27 (n=2 strains), ST244 (n=3), ST253 (n=2), ST313 (n=2), ST390 (n=2), and ST667 (n=2) (see Table S2 Supplementary Material).

### 3.3. Detection of Virulence Markers

The distribution of thirteen genes involved virulence, type III secretion system (T3SS), and quorum sensing system was investigated among the 72* P. aeruginosa* strains, and thirteen different profiles were obtained (Table 1).

The T3SS* exoU* gene was detected in 26* P. aeruginosa* strains and the* exoS* in 47 strains. Both genes were amplified in two strains that were confirmed by three independent experiments and sequencing of the amplicons. On the other hand, neither* exoU* nor* exoS* genes were amplified in one strain that also lacked* exoY, exoT, exoA, rhlI, *and* rhlR *genes. At least one of the genes involved in quorum sensing (*lasI, lasR, rhlI*, or* rhlR*) was not amplified in six strains. However,* lasA*,* lasB*,* aprA,* and* rhlAB* genes were amplified in all strains (Table 1). The* lasR* amplicon sized higher than 2,000 bp in three strains (Pc5, Pc10, and Ps519). After sequencing, the insertion sequences IS*1246*, IS*Ppu17* and IS*1394*, respectively, were detected surrounding* lasR* gene. IS*1246* was detected upstream of* lasR* gene in Pc5 strain. IS*Ppu17* was observed truncating* lasR* gene at position 660 in Pc10 strain, and IS*1394* at position 639 in Ps519 strain; therefore those sequences were submitted in GenBank database with accession numbers MH050328 and MG815635, respectively.

### 3.4. Detection and Characterisation of Integrons

No class 1, 2, or 3 integrases were detected among the 72* P. aeruginosa* strains.

### 3.5. Characterisation of Porin OprD

The* oprD* gene was amplified in all* P. aeruginosa* strains, and their OprD amino acid sequences (443 amino acids) were compared with the OprD sequence of reference* P. aeruginosa* PAO1.  Table 2 shows the polymorphism detected in OprD sequences. Eight OprD profiles were distinguished among the 72* P. aeruginosa* strains, all but one strain being imipenem-susceptible ones. Only four strains had the same pattern as* P. aeruginosa* PAO1, and the pattern B was the most frequently detected (31 strains). Among the amino acid changes detected in OprD loop 1 (44 to 61 amino acid (aa)), the most frequent ones were D43N, S57E, and S59R (31 strains, 43%). Alterations in loop 2 (93 to 127 aa) were observed in 51% of the* P. aeruginosa* strains, T103S and K115T being the most frequent in 21 strains and V127L in 16 strains. The E185Q, P186G, and V189T amino acid changes were the most common described in loop 3 (153 to 192 aa) followed by F170L. On the other hand, E230K change in the loop 4 (221 to 233 aa) was detected in 47 of the strains. The N262T and A267S amino acid changes in loop L5 (260 to 274 aa) and R310E and A315G in loop 6 (304 to 317 aa) were identified. Thirty-one strains showed the G425A or Q424E substitutions in loop 8 (418 to 431 aa). In the region from 372 to 383 aa of the loop 7, the deletion of two amino acids (loop L7-short), which encoded a protein OprD of 441 amino acids, was identified in 46 strains (64%).

Only the imipenem-resistant strain (Pc1) showed a premature stop codon, due to a point mutation which modifies the expected OprD protein (pattern H in Table 2).

A total of 6* P. aeruginosa* strains (Ps514, Ps518, Ps487, Ps499, Ps486, and Ps506) with different protein OprD profiles were selected to study their outer membrane proteins by SDS-PAGE. OprD band was detected in all tested strains.

## 4. Discussion

This study intended to gain new insights into the* P. aeruginosa* in non-clinical environments, analysing the occurrence, and characteristics of* P. aeruginosa* from faecal samples of children. In fact, the occurrence of* P. aeruginosa* isolates in this study (5%) (HSP 6.3% and HLB 4.3%) was in the range detected in other studies (2.6-24%) [3, 7]. The presence of* P. aeruginosa* in stool constitutes a digestive reservoir of the bacteria that may be of importance in* P. aeruginosa* pathophysiology. In our case, there is not any association between diagnosis or clinical symptoms of the children and the* P. aeruginosa* detection.

Additionally, low antimicrobial resistance levels were detected for ceftazidime (8%), cefepime (7%), aztreonam (7%), gentamicin (3%), ciprofloxacin (1%), or imipenem (1%) among the isolates tested. Only four multidrug-resistant strains were found. High susceptibility rates were detected among the* P. aeruginosa* isolates from Logroño (La Rioja). In a previous work performed in the same region by our group, all eight* P. aeruginosa* isolated from healthy humans were susceptible to all tested antibiotics [3]. All these results contrast with the frequent multidrug-resistant* P. aeruginosa* detected in studies carried out with clinical strains [5, 6]. Additionally, in our study, only one imipenem-resistant strain was found and this resistance was associated with the loss of porin OprD by a premature stop codon (277 amino acids). Porin OprD serves as the imipenem entryway, and the presence of premature stop codons or certain insertions/deletions in the* oprD* sequence involves the OprD loss and confers carbapenem resistance in* P. aeruginosa* [1, 6, 16]. On the other hand, a wide variety of amino acid changes were detected in OprD of the remaining 71* P. aeruginosa* strains of our study, even if they were susceptible to imipenem or meropenem. Thirty-one of our strains (43%) contained the same changes in protein OprD (D43N, S57E, S59R, E202Q, I210A, E230K, S240T, N262T, A267S, A281G, K296Q, Q301E, R310G, V359L, and loop L7-short). Some of these amino acid changes had been shown by other authors, in both carbapenem-susceptible and -resistant strains [19–22]. It is known that L2 and L3 loops are involved in binding to imipenem [23–25]. However, most of our strains showed substitutions in both loops and were carbapenem-susceptible, as other studies previously described [22]. The deletion of two amino acids, named loop L7-short, identified in 48 of these strains has been previously described in both MBL-producing and non-MBL-producing isolates [6]. Summarising, there is a high polymorphism in the* oprD* gene of our* P. aeruginosa* strains, but neither the loop L7-short nor amino acid changes, with the exception of the premature stop codon, were associated with carbapenem resistance.

Regarding molecular typing, a wide variety of sequence types has been found in this study (41 different ST among the 48* P. aeruginosa* strains tested), in contrast to other studies with clinical* P. aeruginosa* isolates, in which mainly appeared the “high-risk clones” ST111, ST175, and ST235. Some works showed that a high genetic diversity is associated with low rates of antimicrobial resistance, as our work has also demonstrated [8, 26]. So, the spread of these different STs among the community is independent from the antimicrobial resistance, but other features could be involved such as host adaptation (virulence, biofilm activity…). Among the diversities of STs found in this work, intercontinental clones were encountered: ST27, ST244, ST274, ST277, ST395, ST446, and ST560 [27]. ST244 that was detected in three strains from both regions is a global* P. aeruginosa* clone identified in several countries, among different origins (clinical, animal, and environmental), and associated with different antimicrobial resistance phenotypes (MDR or susceptible strains) and genotypes (carbapenemase-positive or negative) [27–30].* P. aeruginosa *belonging to ST313 and ST274 have been already described as intestinal colonisers in healthy humans [8], although ST274 has been also previously described in carbapenem-susceptible* P. aeruginosa* isolates [3, 8], associated with cystic fibrosis patients, and is considered as an epidemic clone circulating in Spain [29, 31, 32].* P. aeruginosa* strains ascribed to ST27, ST252, ST253 (clonal complex PA14), ST395, and ST560 have been previously observed in clinical animal and environmental samples [14, 27, 31, 33, 34].

Considering* P. aeruginosa* pathogenicity, this species possesses at least two well-defined and interrelated quorum sensing (QS) systems,* las* and* rhl*. These signalling mechanisms are used to regulate gene expression through the production and secretion of autoinducers called N-acylhomoserine lactones, produced by* lasI* and* rhlI* genes, which activate LuxR family regulators, LasR and RhlR, respectively [35]. These systems control the production of different virulence factors, including elastases (LasB and LasA), alkaline protease (AprA), hydrogen cyanide, exotoxin A, pyocyanin, lectins, rhamnolipids (RhlA and RhlB), and superoxide dismutase [36–38]. In our study, the genes implicated in QS system,* las* and* rhl*, were amplified in most of the* P. aeruginosa* isolates. However,* rhlI* and* rhlR* genes were absent in one strain,* rhlR* gene were in other three strains, and* lasI* and* lasR* genes were not amplified in two more strains. Moreover, the insertion sequence IS*1394* was found truncating* lasR* gene in one* P. aeruginosa* strain. Several reports have mentioned the frequency of* lasR* mutations among clinical and environmental isolates [39, 40] and have demonstrated that a* lasR* mutation does not lead to loss of virulence factors thanks to the regulation mediated by* rhlR* gene [41].

One important determinant of virulence is the T3SS, which is present in several Gram-negative bacilli, including* Salmonella*,* Shigella*, and* Yersinia* spp. In* P. aeruginosa*, the T3SS is a major virulence weapon that contributes to cytotoxicity and acute infections [42].* P. aeruginosa* is able to produce and secrete virulence factors directly. This secretion system injects potent ExoS and ExoT (two ADP-ribosylatin enzymes), ExoU (acute cytolytic factor), and ExoY (an adenilate cyclase) exotoxins into the cytoplasm of the host cells by a cell contact-mediated mechanism [43–48]. ExoU is one of the most important virulence determinants of* P. aeruginosa* and is associated with an increased risk of early clinical mortality [49–51]. In our study, two strains showed the infrequent* exoU/exoS*-positive genotype, which has been rarely reported [52, 53]. Additionally, other 24 of our strains amplified the* exoU* gene (33% of total) that belonged to important clinical STs, such as ST253 or ST560, among others. This is a high percentage of* exoU* presence, considering that these strains were recovered from healthy children. Defined clonal lineages are expected to be linked to specific T3SS genotypes. Indeed, in a Spanish multicenter study, the vast majority of the extensively drug resistant (XDR) isolates belonged to the high-risk clones ST175 and ST111, which showed in all cases an* exoU*-negative/*exoS*-positive genotype, and to the ST235 clone that showed an* exoU*-positive/*exoS*-negative genotype [54].

## 5. Conclusion

This work assesses for the first time occurrence of* P. aeruginosa* in faecal samples of children from two different geographical Spanish regions, providing fundamental information about antimicrobial resistance, virulence, and the genetic lineages of the recovered isolates. These faecal* P. aeruginosa* isolates were characterised by their high clonal diversity, great variety of genetic lineages, low antimicrobial resistance percentages, and variability of virulence patterns. Intestinal colonisation by* P. aeruginosa*, mainly belonging to international clones (such as ST244, ST253, or ST274), is an important factor for the development of systemic infections and the environmental dissemination. Periodic active surveillance is useful to identify these community human reservoirs and control the evolution of antibiotic resistance carriage and virulence activity over time.

## Figures and Tables

**Table 1:** Virulence patterns detected among the 72 *P. aeruginosa* isolated from faecal samples of children.

**No. strains**	**Amplification of genes**													**Virulence pattern**
	***exoU***	***exoS***	***exoY***	***exoT***	***exoA***	***lasA***	***lasB***	***aprA***	***rhlAB***	***rhlI***	***rhlR***	***lasI***	***lasR***	
39	-	+	+	+	+	+	+	+	+	+	+	+	+	I
1	-	+	+	+	+	+	+	+	+	+	+	+	+^a^	II
1	-	+	-	+	+	+	+	+	+	+	+	+	+	III
1	-	+	+	-	+	+	+	+	+	+	+	+	+	IV
1	-	+	+	+	+	+	+	+	+	+	-	+	+	V
2	-	+	+	+	+	+	+	+	+	+	+	-	-	VI
14	+	-	+	+	+	+	+	+	+	+	+	+	+	VII
1	+	-	+	+	+	+	+	+	+	+	+	+	+^b^	VIII
6	+	-	-	+	+	+	+	+	+	+	+	+	+	IX
1	+	-	-	+	+	+	+	+	+	+	+	+	+^c^	X
2	+	-	-	+	+	+	+	+	+	+	-	+	+	XI
2	+	+	+	+	+	+	+	+	+	+	+	+	+	XII
1	-	-	-	-	-	+	+	+	+	-	-	+	+	XIII

^a^  *lasR *gene truncated by IS*Ppu17* element

^b^  *lasR *gene truncated by IS*1394* element

^c^  *lasR *gene truncated by IS*1246* element

**Table 2:** Polymorphisms detected in porin OprD of the 72 *P. aeruginosa* isolates from faecal samples of children.

**Strains (n**° **total)**_ _^**a**^	**OprD Size**	**OprD Pattern**	**Amino acid Changes**
Ps514, Pc6, Pc10, Pc15 (4)	443	A	WILD
Ps488, Ps492, Ps493, Ps494, Ps495, Ps496, Ps497, Ps501, Ps502, Ps507, Ps510, Ps515, Ps516, Ps518, Ps520, Ps521, Pc3, Pc8, Pc11, Pc12, Pc13, Pc17, Pc19, Pc22, Pc24, Pc26, Pc27, Pc28, Pc35, Pc39, Pc43 (31)	441	B	D43N; S57E; S59R; E202Q; I210A; E230K; S240T; N262T; A267S; A281G; K296Q; Q301E; R310G; V359L; Loop L7-short
Ps487, Ps498, Ps500, Ps517, Ps519, Ps522, Pc4, Pc5, Pc7, Pc14, Pc18, Pc20, Pc29, Pc30, Pc40, Pc42 (16)	443	C	T103S; K115T; F170L; E185Q; P186G; V189T; R310E; A315G; G425A
Ps489, Ps491, Ps499, Ps505, Ps512, Pc2, Pc23, Pc33, Pc37, Pc38, Pc41 (11)	441	D	S57E; S59R; V127L; E185Q; P186G; V189T; E202Q; I210A; E230K; S240T; N262T; T276A; A281G; K296Q; Q301E; R310E; A315G; L347M; Loop L7-short; S403A; Q424E
Pc16, Pc31, Pc32, Pc36 (4)	441	E	V127L; E185Q; P186G; V189T; E202Q; I210A; E230K; S240T; N262T; T276A; A281G; K296Q; Q301E; R310E; G312R; A315G; L347M; Loop L7-short; S403A; Q424E
Ps486, Pc9, Pc21, Pc25 (4)	443	F	T103S; K115T; F170L
Ps506 (1)	443	G	D43N; T103S; K115T; F170L
Pc1 (1)	277	H	G60R; T105A; V127L; E185Q; P186G; V189T; E202Q; I210A; E230K; S240T; S248E; N262T; A267S; W277STOP

## Data Availability

All data are included in the manuscript and Supplementary Material. The new sequence detected of IS*1394* truncating* lasR* gene in Ps519 strain was submitted in GenBank database with accession number MG815635.

## Supplementary Materials

Supplementary Materials Table S1: primers and annealing temperatures used in the PCR reactions of this study. Table S2: characteristics of* P. aeruginosa* isolates recovered from faecal samples of children.
