# Supplementary material for: Pseudomonas aeruginosa Isolates from Spanish Children: Occurrence in Faecal Samples, Antimicrobial Resistance, Virulence, and Molecular Typing
Source: Biomed Res Int. 2018 Jun 11;2018:8060178. doi: 10.1155/2018/8060178 (PMC6016177; doi:10.1155/2018/8060178)
Supplement: Supplementary Materials — Table S1: primers and annealing temperatures used in the PCR reactions of this study. Table S2: characteristics of P. aeruginosa isolates recovered from faecal samples of children. [file 8060178.f1.pdf]

Supplementary Material.

Table S1: Primers and annealing temperatures used in the PCR reactions of this study.

Primer name	Sequence (5' → 3')	Target gene	PCR product size (bp)	Annealing temperature (°C)	Reference
acsA-F	ACCTGGTGTACGCCTCGCTGAC	*acsA*	842	54	1
acsA-R	GACATAGATGCCCTGCCCCTTGAT				
aroE-F	TGGGGCTATGACTGGAAACC	*aroE*	1053	55	1
aroE-R	TAACCCGGTTTTGTGATTCCTACA				
guaA-F	CGGCCTCGACGTGTGGATGA	*guaA*	940	56	1
guaA-R	GAACGCCTGGCTGGTCTTGTGGTA				
mutL-F	CCAGATCGCCGCCGGTGAGGTG	*mutL*	940	58	1
mutL-R	CAGGGTGCCATAGAGGAAGTC				
nuoD-F	ACCGCCACCCGTACTG	*nuoD*	1042	55	1
nuoD-R	TCTCGCCCATCTTGACCA				
ppsA-F	GGTCGCTCGGTCAAGGTAGTGG	*ppsA*	989	56	1
ppsA-R	GGGTTCTCTTCTTCCGGCTCGTAG				
trpE-F	GCGGCCCAGGGTCGTGAG	*trpE*	811	56	1
trpE-R	CCCGGCGCTTGTTGATGGTT				
exoU-F	ATGCATATCCAATCGTTG	*exoU*	2000	55	2
exoU-R	TCATGTGAACTCCTTATT				
exoS-F	GCGAGGTCAGCAGAGTATCG	*exoS*	118	60	2
exoS-R	TTCGGCGTCACTGTGGAT				
exoY-F	CGGATTCTATGGCAGGGAGG	*exoY*	289	60	2
exoY-R	GCCCTTGATGCACTCGACCA				
exoT-F	AATCGCCGTCCAACTGCATGCG	*exoT*	152	60	2
exoT-R	TGTTCGCCGAGGTACTGCTC				
exoA-F	GACAACGCCCTCAGCATCACCAGC	*exoA*	396	60	2

exoA-R	CGCTGGCCCATTCGCTCCAGCGCT				
lasA-F	CGCCATCCAACCTGATGCAAT	*lasA*	514	60	2
lasA-R	AGGCCGGGGTTGTACAACGGA				
lasB-F	TTCTACCCGAAGGACTGATAC	*lasB*	153	55	2
lasB-R	AACACCCATGATCGCAAC				
aprA-F	ACCCTGTCCTATTCGTTCC	*aprA*	140	55	2
aprA-R	GATTGCAGCGACAACTTGG				
rhlAB-F	TCATGGAATTGTCACAACCGC	*rhlAB*	151	60	2
rhlAB-R	ATACGGCAAAATCATGGCAAC				
rhlI-F	CTTGGTCATGATCGAATTGCTC	*rhlI*	625	60	2
rhlI-R	ACGGCTGACGACCTCACAC				
rhlR-F	CAATGAGGAATGACGGAGGC	*rhlR*	730	60	2
rhlR-R	GCTTCAGATGAGGCCCAGC				
lasI-F	ATGATCGTACAAATTGGTCGGC	*lasI*	605	60	2
lasI-R	GTCATGAAACCGCCAGTCG				
lasR-F	CGGGTATCGTACTAGGTGCATCA	*lasR*	1100	60	2
lasR-R	GACGGGAAAGCCAGGAAACTT				
intI1-F	GGGTCAAGGATCTGGATTTCG	*intI1*	483	62	3
intI1-R	ACATGCGTGTAAATCATCGTCG				
intI2-F	CACGGATATGCGACAAAAAGGT	*intI2*	788	62	3
intI2-R	GTAGCAAACGAGTGACGAAATG				
intI3-F	GCCTCCGGCAGCGACTTTCAG	*intI3*	979	62	3
intI3-R	ACGGATCTGCCAAACCTGACT				
qacEΔ1-F	GGCTGGCTTTTTCTTGTTATCG	*qacEΔ1-sul1*	1125	63	3
sul1-R	GCGAGGGTTTCCGAGAAGGTG				
OprDlong-F	CTACGCAGATGCGACATGC	*oprD*	1586	55	4

OprDlong-R	CCTTTATAGGCGCGTTGCC				
OprD-F	CGCCGACAAGAAGAACTAGC	*oprD*	1413	55	5
OprD-R	GTCGATTACAGGATCGACAG				

1. Curran B, Jonas D, Grundmann H, Pitt T, Dowson CG (2004) Development of a multilocus sequence typing scheme for the opportunistic pathogen *Pseudomonas aeruginosa*. J Clin Microbiol 42:5644–5649.

2. Petit SM, Lavenir R, Colinon-Dupuich C, Boukerb AM, Cholley P, Bertrand X, Freney J, Doléans-Jordheim A, Nazaret S, Laurent F, Cournoyer B (2013) Lagooning of wastewaters favors dissemination of clinically relevant *Pseudomonas aeruginosa*. Res Microbiol 164(8):856-866.

3. Sáenz Y, Briñas L, Domínguez E, Ruiz J, Zarazaga M, Vila J, Torres C (2004) Mechanisms of resistance in multiple-antibiotic-resistant *Escherichia coli* strains of human, animal and food origins. Antimicrob Agents Chemother 48:3996–4001.

4. Wolter DJ, Hanson ND, Lister PD (2004) Insertional inactivation of *oprD* in clinical isolates of *Pseudomonas aeruginosa* leading to carbapenem resistance. FEMS Microbiol Lett 236:137–143.

5. Gutiérrez O, Juan C, Cercenado E, Navarro F, Bouza E, Coll P, Pérez JL, Oliver A (2007) Molecular epidemiology and mechanisms of carbapenem resistance in *Pseudomonas aeruginosa* isolates from Spanish hospitals. Antimicrob Agents Chemother 51:4329-4335.

Table S2. Characteristics of *P. aeruginosa* isolates recovered from faecal samples of children

Children			Strain[b]	Phenotype[a]	Patterns			
Individual (underlying diseases[a])	Gender[a]	Age (years)			PFGE	MLST[a]	OprD[d]	Virulence[e]
1 (none)	M	13	**Ps514**	Susceptible	21	ST244	A	I
2 (none)	M	4	**Pc6**	Susceptible	35	-	A	I
3 (none)	M	7	**Pc10**	AZT,CAZ, FEP	39	ST244	A	II
4 (none)	M	13	**Pc15**	Susceptible	44	ST244	A	I
5 (atopic dermatitis)	M	1	**Ps488**	Susceptible	3	ST903	B	I
6 (none)	M	10	**Ps492**	Susceptible	6	ST132	B	I
7 (none)	M	1	**Ps493**	Susceptible	7	ST555	B	I
8 (weight delay)	M	3	**Ps494**	Susceptible	8	ST252	B	I
9 (none)	M	5	**Ps495**	Susceptible	9	ST27	B	I
10 (none)	F	2	**Ps496**	Susceptible	10	ST390	B	I
11 (none)	M	5	**Ps497**	Susceptible	10	ST390	B	I
12 (none)	F	11	**Ps501**	Susceptible	14	ST2241[c]	B	I
13 (lactose intolerance)	F	1	**Ps502**	Susceptible	15	ST980	B	I
14 (none)	M	3	**Ps507**	Susceptible	18	ST139	B	I
15 (none)	M	2	**Ps510**	Susceptible	19	ST1205	B	III
16 (none)	F	5	**Ps515**	Susceptible	22	ST668	B	I
17 (none)	M	7	**Ps516**	Susceptible	23	ST1033	B	I
18 (none)	M	1	**Ps518**	Susceptible	25	ST27	B	I
19 (none)	M	2	**Ps520**	Susceptible	27	ST395	B	I
20 (none)	F	3	**Ps521**	Susceptible	28	ST499	B	I
21 (none)	M	8	**Pc3**	Susceptible	32	ST527	B	I
22 (none)	F	13	**Pc8**	Susceptible	37	-	B	I
23 (Williams syndrome)	F	0.25	**Pc11**	GEN	40	-	B	IV
24 (none)	F	2	**Pc12**	Susceptible	41	-	B	I
25 (none)	F	2	**Pc13**	Susceptible	42	-	B	I
26 (none)	M	5	**Pc17**	Susceptible	46	ST508	B	I

Children			Strain[b]	Phenotype[a]	Patterns			
Individual (underlying diseases[a])	Gender[a]	Age (years)			PFGE	MLST[a]	OprD[d]	Virulence[e]
27 (none)	F	2	**Pc19**	Susceptible	48	-	B	I
28 (none)	F	9	**Pc22**	Susceptible	51	ST254	B	I
29 (none)	M	9	**Pc24**	Susceptible	53	-	B	V
30 (none)	F	2	**Pc26**	Susceptible	55	-	B	I
31 (anorexia)	F	13	**Pc27**	Susceptible	56	-	B	I
32 (none)	M	1	**Pc28**	Susceptible	57	-	B	I
33 (none)	M	3	**Pc35**	Susceptible	61	-	B	I
34 (none)	M	0.50	**Pc39**	Susceptible	65	-	B	I
35 (none)	M	4	**Pc43**	TZP, AZT, CAZ, FEP	68	ST274	B	I
36 (none)	M	2	**Ps487**	Susceptible	2	ST446	C	VII
37 (none)	F	0.14	**Ps498**	Susceptible	11	ST253	C	VII
38 (none)	F	0.11	**Ps500**	Susceptible	13	ST299	C	I
39 (weight delay)	M	0.06	**Ps517**	Susceptible	24	ST1197	C	VII
40 (atopic dermatitis)	F	1	**Ps519**	Susceptible	26	ST253	C	VIII
41 (none)	M	3	**Ps522**	Susceptible	29	ST1664	C	I
42 (none)	F	10	**Pc4**	Susceptible	33	ST1648	C	VI
43 (none)	F	13	**Pc5**	Susceptible	34	-	C	X
44 (cystic fibrosis)*	F	4	**Pc7**	TZP	36	-	C	VII
45 (none)	M	1	**Pc14**	Susceptible	43	-	C	VII
46 (none)	M	0.50	**Pc18**	Susceptible	47	ST2222[c]	C	XII
47 (none)	F	7	**Pc20**	Susceptible	49	-	C	I
48 (none)	M	5	**Pc29**	Susceptible	58	ST2223[c]	C	VII
49 (none)	M	0.33	**Pc30**	GEN, CIP	58	-	C	VII
50 (none)	F	2	**Pc40**	TZP, AZT, CAZ, FEP	66	-	C	I
51 (none)	M	7	**Pc42**	Susceptible	67	-	C	VII
52 (PD/D)	M	3	**Ps489**	Susceptible	4	ST1182	D	IX
53 (none)	M	2	**Ps491**	Susceptible	5	ST671	D	VII
54 (lactose intolerance)	F	0.33	**Ps499**	Susceptible	12	ST207	D	VII
55 (none)	F	1	**Ps505**	Susceptible	16	ST2242[c]	D	IX

Children			Strain[b]	Phenotype[a]	Patterns			
Individual (underlying diseases[a])	Gender[a]	Age (years)			PFGE	MLST[a]	OprD[d]	Virulence[e]
56 (none)	F	2	**Ps512**	Susceptible	20	ST560	D	VII
57 (none)	F	11	**Pc2**	Susceptible	31	ST1149	D	VII
58 (none)	M	1	**Pc23**	Susceptible	52	ST313	D	VII
59 (none)	M	1	**Pc33**	TZP, AZT, CAZ, FEP	60	ST1411	D	VII
60 (none)*	M	0.67	**Pc37**	Susceptible	63	ST313	D	XI
61 (none)	M	8	**Pc38**	Susceptible	64a	-	D	IX
62 (none)	F	1	**Pc41**	Susceptible	64b	-	D	IX
63 (none)	F	10	**Pc16**	Susceptible	45	ST2143	E	XII
64 (none)	M	1	**Pc31**	TZP, AZT, CAZ, FEP	59	ST667	E	IX
65 (none)	M	12	**Pc32**	Susceptible	59	-	E	IX
66 (none)	F	2	**Pc36**	Susceptible	62	ST667	E	XI
67 (none)	F	5	**Ps486**	Susceptible	1	ST277	F	I
68 (none)	M	2	**Pc9**	Susceptible	38	ST2125	F	I
69 (none)	M	2	**Pc21**	Susceptible	50	-	F	I
70 (none)	M	5	**Pc25**	Susceptible	54	-	F	VI
71 (none)	F	14	**Ps506**	CAZ	17	ST1398	G	I
72 (none)	F	1	**Pc1**	IPM	30	ST2221[c]	H	XIII

[a] Abbreviations: PD/D, psychomotor disturbance / delay; M, male; F, female; AZT, aztreonam; CAZ, ceftazidime; FEP, cefepime; GEN, gentamicin; TZP, piperacillin-tazobactam; IPM, imipenem; -, not determined. * This child had received antibiotic treatment in the three months previous to sampling.

[b] Pc are strains from HLB; and Ps from HSP

[c] New MLST detected

[d] OprD patterns classified according to Table 2.

[e] Virulence profiles defined according to Table 1.
